# MicroRNAs and their targets in cucumber shoot apices in response to temperature and photoperiod

**DOI:** 10.1186/s12864-018-5204-x

**Published:** 2018-11-15

**Authors:** Xiaohui Zhang, Yunsong Lai, Wei Zhang, Jalil Ahmad, Yang Qiu, Xiaoxue Zhang, Mengmeng Duan, Tongjin Liu, Jiangping Song, Haiping Wang, Xixiang Li

**Affiliations:** 1 Key Laboratory of Biology and Genetic Improvement of Horticultural Crops, Ministry of Agriculture; Institute of Vegetables and Flowers, Chinese Academy of Agricultural Sciences, Beijing, 100081 China; 2 Institute of Pomology & Olericulture, Sichuan Agricultural University, Chengdu, 611130 China

**Keywords:** Cucumber, Shoot apex, miRNAs, Photoperiod, Temperature

## Abstract

**Background:**

The cucumber is one of the most important vegetables worldwide and is used as a research model for study of phloem transport, sex determination and temperature-photoperiod physiology. The shoot apex is the most important plant tissue in which the cell fate and organ meristems have been determined. In this study, a series of whole-genome small RNA, degradome and transcriptome analyses were performed on cucumber shoot apical tissues treated with high vs. low temperature and long vs. short photoperiod.

**Results:**

A total of 164 known miRNAs derived from 68 families and 203 novel miRNAs from 182 families were identified. Their 4611 targets were predicted using psRobot and TargetFinder, amongst which 349 were validated by degradome sequencing. Fourteen targets of six miRNAs were differentially expressed between the treatments. A total of eight known and 16 novel miRNAs were affected by temperature and photoperiod. Functional annotations revealed that “Plant hormone signal transduction” pathway was significantly over-represented in the miRNA targets. The miR156/157/*SBP-Boxes* and novel-mir153/*ethylene-responsive transcription factor/senescence-related protein/aminotransferase/acyl-CoA thioesterase* are the two most credible miRNA/targets combinations modulating the plant’s responsive processes to the temperature-photoperiod changes. Moreover, the newly evolved, cucumber-specific novel miRNA (novel-mir153) was found to target 2087 mRNAs by prediction and has 232 targets proven by degradome analysis, accounting for 45.26–58.88% of the total miRNA targets in this plant. This is the largest sum of genes targeted by a single miRNA to the best of our knowledge.

**Conclusions:**

These results contribute to a better understanding of the miRNAs mediating plant adaptation to combinations of temperature and photoperiod and sheds light on the recent evolution of new miRNAs in cucumber.

**Electronic supplementary material:**

The online version of this article (10.1186/s12864-018-5204-x) contains supplementary material, which is available to authorized users.

## Background

Temperature and photoperiod are two of the most important factors affecting plant development [1–3]. The cucumber is an important vegetable worldwide and is extensively produced via protected cultivation. Temperature and photoperiod can affect plant architecture, floral bud differentiation, sex expression, stress tolerance, fruit productivity, phytochemicals accumulation, flavours and nutrient formation in cucumbers [4–7]. Therefore, revealing the molecular mechanisms underlying the developmental modification in response to temperature and photoperiod changes will contribute substantially to cucumber production.

The plant genome responds to environments via epigenetic, transcriptional and post-transcriptional regulations [8]. The epigenetic modifications include chromosome organization, DNA methylation and histone modifications [9, 10]. DNA methylation changes have been revealed in cucumber shoot apexes grown in differential temperatures in previous studies [11]. The DNA methylation patterns also changed in male gametophytes of cucumber plants infected by viroids [12]. Transcriptional regulation of genes encoding photosynthetic enzymes, H^+^-ATPase, leaf volatile compounds, Argonaute, Dicer-like, RNA-dependent RNA polymerase, NAC transcription factors and superoxide dismutase (SOD) have been proven to play roles in suboptimal temperature and light adaptation and abiotic stress tolerance in cucumbers [13–19]. Transcriptome sequencing has been performed on cucumbers to analyse the transcripts responsible for sex expression, fruit length, waterlogging reaction and *Phytophthora capsici* resistance [20–23]. From the post-transcriptional control level, non-coding small RNA is the best-studied mechanism till now. Small RNAs not only play fundamental roles in post-transcriptional regulation via mRNA cleavage and translation inhibition but also link up these three levels of regulation in the way of epigenetic reprogramming [24, 25].

MicroRNAs (miRNAs) are a class of well-studied small non-coding RNAs. In plants, miRNAs are generated from primary miRNA transcripts (pri-miRNAs) containing a distinctive hairpin structure, which are first trimmed by DICER-LIKE1 (DCL1) to generate miRNA precursors (pre-miRNAs) in the nucleus and then transported to the cytoplasm and further processed to generate ~ 21 nt mature miRNAs [26, 27]. Subsequently, the mature miRNAs are loaded onto the RNA-induced silencing complex (RISC) and guide transcript cleavage (Bartel, 2004; Baulcombe, 2004) or translational repression of their target mRNAs [25, 28]. Plant miRNAs are highly complementary to their targets, which make it possible to predict and verify their targets using bioinformatics and degradome sequencing methods.

miRNAs play important roles in a wide range of plant developmental processes, including plant architecture [29], leaf morphology [30], root development [31], tuberization [32], the vegetative-reproductive phase change [33], floral organ identity [28, 34], flowering time [35], self-incompatibility [36], cytoplasmic male sterility [37], plant nutrient homeostasis [38], and response to biotic and abiotic stresses [39–41]. In cucumbers, the miRNAs derived from leaf, root, stem and phloem exudate were analysed in three independent high-throughput sequencing studies [42–44]. However, neither miRNAs in the shoot apex nor the miRNAs at different temperature and photoperiod changes were profiled in cucumber plants.

In cucumbers, approximately 10–15 axillary buds or primordia are formed at the shoot apex in seedlings. The environmental conditions of the seedling stage determine the lateral organs such as male or female flowers in the formation of the next 10–15 nodes. Thus, the performances of genes in the cucumber shoot apexes are particularly significant from a horticulture perspective.

In this study, the miRNAs in cucumber shoot apexes were identified. The expression profiles from short and long photoperiods, low and high temperatures were compared, by a series of small RNA sequencing experiments. The targets of these miRNAs were predicted and proven by degradome sequencing. The expression levels of the predicted targets were also profiled by transcriptome sequencing. Based on these data, the roles of miRNAs on the cucumber adaptation to environmental temperature and photoperiod changes were investigated. A newly evolved cucumber specific novel-miRNA and its evolutionary path were highlighted.

## Results

### sRNA sequencing and miRNA identification

To reveal the roles of miRNAs in plant adaptation to temperature-photoperiod environments, small RNAs were pyrosequenced from the shoot apical tissues of cucumber plants grown in four differential temperature-photoperiod treatments (16 h light at 28 °C /8 h dark at 25 °C, HL; 8 h light at 28 °C /16 h dark at 25 °C, HS; 16 h light at 20 °C /8 h dark at 15 °C, LL; 8 h light at 20 °C /16 h dark at 15 °C, LS). The 16 h/8 h and 8-h/16 h of light/dark photoperiods simulated long- and short-day seasons. The 28 °C/25 °C and 20 °C/15 °C conditions were used to simulate the high and low temperature climates, respectively. A total of 209.25 M reads were produced from the 12 samples (four treatments, three biological replicates). Approximately 16.63 ~ 17.65 M high quality clean reads were obtained for each sample (Additional file 1: Table S1). The majority of the tags ranged in size between 18 and 28 nt, with the 24 nt and 21 nt lengths dominating (Additional file 2: Figure S1). The total reads were converted to non-redundant unique tags and then were aligned to GenBank, Rfam and the cucumber genome. The reads annotated as potential miRNAs and those derived from intronic regions and exon antisense regions as well as the unannotated reads were used for miRNA mining based on the secondary structure (hairpin folding) of their precursors and other criteria defined in the Methods section. The miRNA candidates were aligned to all known plant miRNAs in miRBase 21.0, allowing two-base mismatch and a two-nucleotide overhang. The matched miRNAs were termed as known miRNAs and named after their best-match families. The members within the same miRNA family were alphabetical labelled according to their genome location. A total of 68 known miRNA families containing 164 members were identified (Table 1). The largest family was miR7129, which contains 13 members, followed by miR169, which harbours 12 members. Both 5p and 3p miRNAs were detected for 76 precursors, while the remaining 88 precursors had only one mature miRNA for each. Detailed information, including nomenclature, genome location, mfe, sequences and counts of 5p and 3p mature miRNAs, precursor sequences and their secondary structures are listed in (Additional file 1: Table S2). The remaining 203 miRNAs that could not be incorporated into the known families were termed novel miRNAs. Amongst these, 31 novel-mirs were merged into 10 novel families and the other 172 were single copy miRNA genes (Additional file 1: Table S3). Out of these, 19 novel miRNAs contained both 5p and 3p sequences (Table 2).

**Table 1:** Known miRNAs in cucumber shoot tips

miRNA	Family member^a^	Dominant sequence (5p)	count(5p)	Dominant sequence (3p)	count(3p)	Previously identified		
csa-miR156	7	TGACAGAAGAGAGTGAGCAC	407,153	TGCTCACTTCTCTTTCTGTCAGC	128	M	Y	X
csa-miR157	4	TTGACAGAAGATAGAGAGCAC	2,059,132	GCTCTCTATGCTTCTGTCATC	158		Y	X
csa-miR159	1	–	–	ATTGGATTGAAGGGAGCTCC	14	M	Y	X
csa-miR160	3	TGCCTGGCTCCCTGTATGCCA	11,461	GCGTATGAGGAGCCAAGCATA	24,040	M	Y	X
csa-miR162	1	GGAGGCAGCGGTTCATCGACC	318	TCGATAAACCTCTGCATCCAG	3306		Y	X
csa-miR164	5	TGGAGAAGCAGGGCACGTGCA	253,447	CACGTGCTCCCTTTCTCCAAC	1649	M	Y	X
csa-miR166	8	GGAATGTTGTCTGGCTCGAGG	92,459	TCGGACCAGGCTTCATTCCCC	3,561,572	M	Y	X
csa-miR167	6	TGAAGCTGCCAGCATGATCTA	2,016,681	GATCATATGGTAGCTTCACC	34	M	Y	X
csa-miR168	2	TCGCTTGGTGCAGGTCGGGAA	172,731	TCCCGCCTTGCATCAACTGAA	17,238	M	Y	X
csa-miR169	12	AAGCCAAGGATGAATTGCCGG	35,494	GGCAATTCCATTCTTGGCTAAG	511	M	Y	X
csa-miR171	7	TATTGGTCCGGTTCACTCAGA	1589	TGATTGAGCCGCGCCAATATC	4086	M	Y	X
csa-miR172	5	GCGGCATCATCAAGATTCACA	3255	AGAATCTTGATGATGCTGCAT	132,778	M	Y	X
csa-miR390	4	AAGCTCAGGAGGGATAGCGCC	174,310	CGCTATCCATCCTGAGTTTCA	1220	M	Y	X
csa-miR393	3	TCCAAAGGGATCGCATTGATC	146	ATCGTGCGATCCCTTAGGAAT	11	M	Y	X
csa-miR394	2	TTGGCATTCTGTCCACCTCC	1749	AGGTGGGCATACTGCCAATTG	11		Y	X
csa-miR395	4	GTTCCCTTCCAAACACTTCAGA	1	TGAAGTGTTTGGGGGAACTC	33			X
csa-miR396	5	TTCCACAGCTTTCTTGAACTT	223	GCTCAAGATAGCTGTGGGAAA	1433	M	Y	X
csa-miR397	1	TCATTGAGTGCAGCGTTGATG	188	–	–	M	Y	X
csa-miR398	1	GAGTGAACCTGAGAACACAAGA	15,821	TTGTGTTCTCAGGTCACCCCT	105	M	Y	X
csa-miR399	6	GGGCGGATCTTTTTTGGCAGG	239	TGCCAAAAGAGACTTGCCCTG	6739	M	Y	X
csa-miR408	1	GCGGGGAACAGACAGAGCATG	2242	ATGCACTGCCTCTTCCCTGGC	352	M	Y	X
csa-miR477	2	TTCTCTCCCTCAAGGGCTTCGA	324	GAAGCCCACGAGGAGGGGATG	228		Y	
csa-miR530	2	TGCATTTGCACCTACACCTTC	3949	–	–		Y	X
csa-miR827	1	–	–	TTAGATGACCATCAACGAACG	90	M	Y	X
csa-miR860	1	–	–	TCAAAGATTGGACAAAATTAT	12			
csa-miR1038	1	CAATGGTGGAATCGATCAGGC	7	–	–			
csa-miR1041	5	–	–	TTTGTACGGGTGAGAGTGGTCAG	28			
csa-miR1044	3	–	–	TTGAATAATGCATATTTTTTT	7			
csa-miR1120	1	TAAGTATAAGATCGTTTAGAC	14	–	–			
csa-miR1886	1	–	–	TGAAGAAGTAGATGAAGAGTC	170			
csa-miR1888	1	TTATTAAGATTGTTTGAAGAA	7	–	–			
csa-miR2111	2	TAATCTGCATCCTGAGGTTTA	14,800	GTCCTTGGGATGCAGATTATC	39		Y	X
csa-miR2628	1	CTGAAAGAGAAGATGAATAA	14	–	–			
csa-miR2950	1	TTCCATCTCTTGCACACTGGA	6356	TGGTGTGCATGAGATGGAATA	14,275	M	Y	X
csa-miR3512	1	–	–	TCACAAATGAAGACAATAGA	32			
csa-miR3704	1	–	–	GGTGTACGGTGGAGTGGAAAATA	26			
csa-miR4234	1	–	–	CAAATTAAAATCGTGGGCAT	8			
csa-miR4249	3	–	–	TGAATTTGGAGAAGGAGAGTA	18			
csa-miR4383	1	TATTGGAGCATCAGTGAAACGTC	234	–	–			
csa-miR4414	1	AGCTGCTGACTCGTTGGCTCA	716,542	AACCAACGATGCAGGAGCCAA	123			X
csa-miR5041	1	CTTAGAGCAGATGAAGATGAA	20	–	–			
csa-miR5083	1	AGACTACAATTATCTGATCA	1274	–	–			
csa-miR5225	1	CCGCAGGAGAGATGACACCCAC	14	–	–			
csa-miR5242	1	TTGTAGAAATCAAAGATGACA	7	–	–			
csa-miR5337	1	TTAGGAACGGTAGCAATTTGA	16	–	–			
csa-miR5532	1	TGGAATATATGACAAAGGTGG	59	–	–			
csa-miR5667	1	AAAAGAGATCAAATGGATGCC	13	–	–			
csa-miR5671	1	–	–	TATGGTAGTAGACATCGGGTGAC	11			
csa-miR5747	1	TTAGAATACTCATACATACATTA	8	–	–			
csa-miR5751	1	TTGATGTTGATCAATGGATATTT	174	–	–			
csa-miR5760	1	–	–	TGCTTTAGGATTTGTTTAGGAT	6			
csa-miR5788	2	–	–	TGGATGTGAAGACAGCTAGTA	53			
csa-miR6182	1	TGTAGTTGATGGATGGATGCTTT	12	–	–			
csa-miR6257	1	–	–	TCTTAACTAGTTGAATTATGT	9			
csa-miR6300	1	–	–	GTCGTTGTAGTATAGTGGTA	9345			
csa-miR6421	1	TACGATGAGATTGTAAGGGAG	8	–	–			
csa-miR6426	5	–	–	GTAGAGACATGGAAGTGGAGACA	252			
csa-miR6440	1	GAGTTGATCGAATTTCGTAGTTT	14	–	–			
csa-miR7129	13	TCAAATCTAAACGATCGTTTTTC	137	–	–			
csa-miR7499	1	TATATTTTCGGGTTATTTGGGTT	8	–	–			
csa-miR7741	1	–	–	TGTTATTGTGGAAGTTCTTGA	1286			
csa-miR8125	1	CAGGAGAAGAATGTGAAAAGGTA	64	–	–			
csa-miR8608	1	TTTCTACACGATCGTTTGACATG	6	–	–			
csa-miR8657	1	–	–	TCGTAGAAATTGTAGAAGCAGGC	15			
csa-miR8693	2	–	–	AGATGAAAGAGATAGATGAGCAT	30			
csa-miR9408	1	CAACAATCGTCAGGATAGAAAAT	56	–	–			
csa-miR9759	1	TATGAACCAAGGTAGAATTTAAA	37	–	–			
csa-miR9776	1	–	–	TTCGGACGAGGATGTTAACGG	12			
Total	164		5,992,813		3,781,493			

^a^Detailed information including nomenclatures, genome location, mfe, sequences and counts of 5p and 3p mature miRNA, precursor sequences and their secondary structures are listed in Additional file 1: Table S2. M: previously identified in ref [44]; Y: previously identified in ref [43]; X: previously identified in ref [42]

**Table 2:** Novel miRNAs harbouring both 5p and 3p sequences

miRNA	mfe	seq(5p)	count(5p)	seq(3p)	Count(3p)
csa-novel-mir3	− 24.2	ATCAAGTCAGGATGGCCGAGT	44	TCAAGTTCTGGTCTTCGTGA	16
csa-novel-mir7	− 93.1	TTCGACTGCCTATATCTTTCCTT	5	GGAATGATGTAGCATGAAAG	3
csa-novel-mir12	− 56.9	TTTTTAACAGATTAATTGGACA	2207	ACCAAATTGATTTAAGAGGC	8
csa-novel-mir13	− 18.27	TCGAACATAAAGGAACATGAGTT	22	CCATCATGGTTCTTTTGTTTGGAC	2
csa-novel-mir29	− 41.29	AACTACTATTCATTTCAAAAT	1	TTGGAAATGAATAGTAGTTTA	14
csa-novel-mir33	− 47.8	TACAAGTTATGACTTATGAGT	3	TCATAACTTGTAACTTGTCCG	8
csa-novel-mir36	−29.62	TTTTTGTGATTCGTGGATTTTTC	51	AATATCCACGTATTGATGGAAATT	1
csa-novel-mir42	− 58.2	TGAAGTATGAACTGAAAGGAA	29	CCTTTCAATTCATACTTATTT	9
csa-novel-mir54	− 45.1	TCGGTGGTCGTGTGGTTTCAG	22	AATCACACAACCATCGATC	1
csa-novel-mir62	− 60.5	CATGACCAAAATGCGAACTTA	38	AGTTTGTTTTTTGGTCATGTC	1
csa-novel-mir63	− 31.7	TCTTTAATCAACTCAACCAAA	1	TGGTTGAGCTGATTTAAGGAT	9
csa-novel-mir68	− 40.3	TTTAAAAACCATACCAGAATT	6	TTTTGGCATGGTTTTTAAACC	1
csa-novel-mir75	− 43.8	CGGTGGAGCGATGTTCTTGGACT	1	TTCGAGAAGGGCTTGATTGGT	14
csa-novel-mir89	−42.51	TTGCTGCTCATTCGTTAGTTC	37	TCTAACGATGTAGGAGCAATC	238
csa-novel-mir104	− 46.9	TCGGACATCGGCGACTTGGAA	51,816	TTCCAAGTCCACCCATGCCCGC	15,481
csa-novel-mir106	− 149.6	TAGCTGTTAAATATGAAGACGA	32,337	GTCTTCATATTTAACAGCTAAT	37
csa-novel-mir113	−41.1	TCGCAGAAGAGATGGTGCCGT	4628	GGCATCATATCCTCTGCGCAA	322
csa-novel-mir136	− 66.54	AGTCTACTGTTTCATTCTTGA	3	AAGAATGAAACAGTAGACTCA	147
csa-novel-mir141	− 21.9	TTTGACACGAGCACCTGAAAAAT	4188	TTTTCGGTTTCTCGAGTTACGAAC	3

### Comparative expression patterns of miRNAs in the cucumber shoot tips

The expression levels of known and novel miRNAs were profiled based on tag counts. For multiple precursors sharing the same mature miRNA sequences, only one member of the mature miRNA was used for expression profiling. miR166, miR167 and miR157 were the three highest expressed known miRNAs, which had 200,167-485,949, 91,482–378,695 and 68,360–369,907 copies, respectively, in the cucumber shoot apical tissues (Additional file 1: Table S4). The two highest abundant novel miRNAs were novel-mir166 and novel-mir175, which had 7139–10,992 and 4605–8714 copies, respectively (Additional file 1: Table S5). Another 16 novel miRNAs also had more than 1 thousand copies for each samples. The detailed expression levels of the known and novel miRNAs are listed in Additional file 1: Tables S4 and S5.

**Fig. 1:**
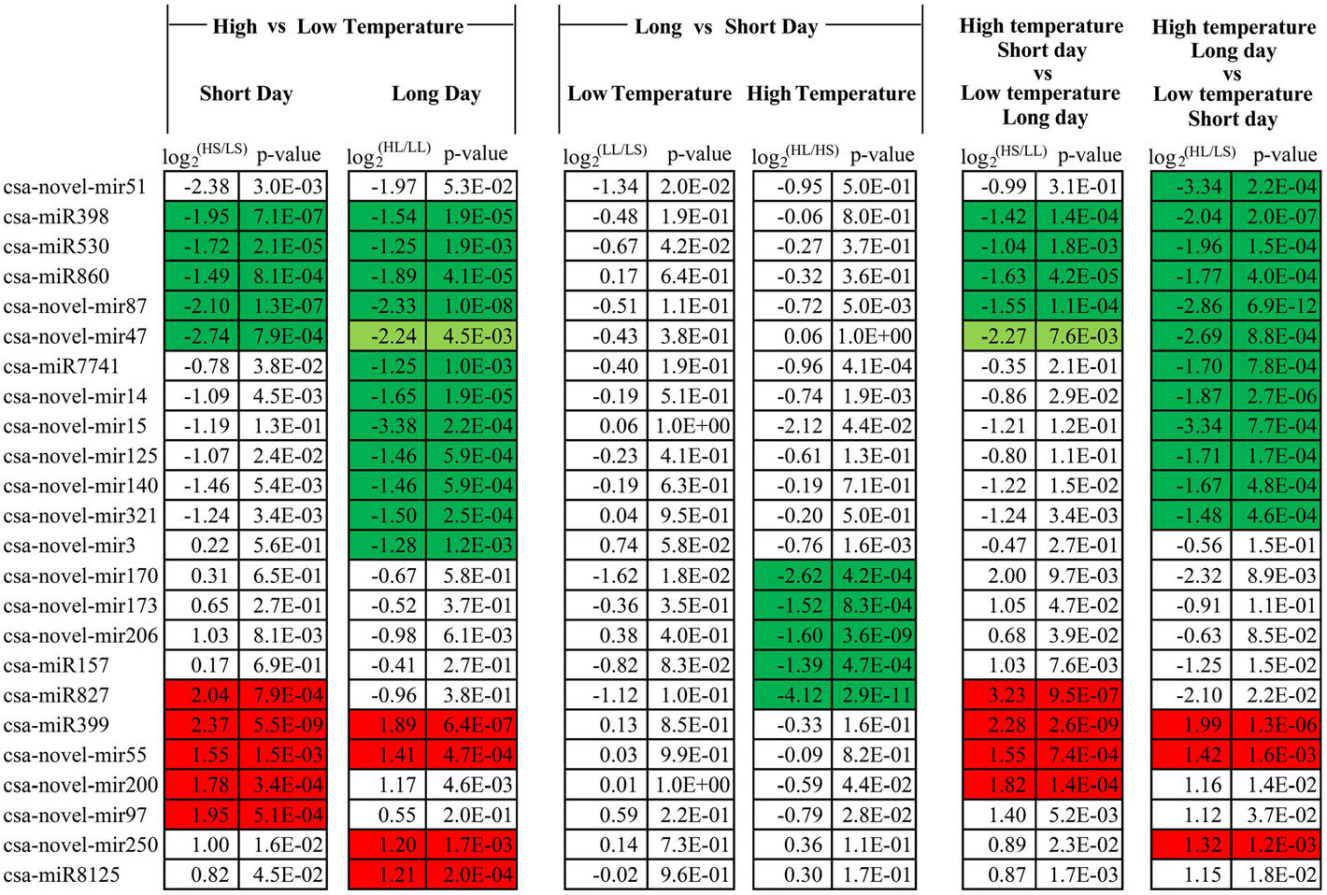
Differentially expressed miRNAs. HS, high temperature, short day; LS, low temperature, short day; HL, high temperature, long day; LL, low temperature, long day. The red shading indicate up-regulated expression; the green shadows indicate down-regulated expression; and the light green shadows indicate down-regulated expression with *p*-value slightly exceeding the 1E-3 level

The expression level between the treatments was compared using normalized tag counts from the three biological replicates. A total of 8 known and 16 novel miRNAs were differentially expressed (more than two-fold change, *p* < 10^− 3^) between at least one pair in an environmental comparison (Fig. 1). Among them, three known miRNAs (miR398, miR530, and miR860) and two novel miRNAs (novel-mir47 and novel-mir87) were suppressed by higher temperature in both short and long photoperiod conditions. One known miRNA (miR7741) and six novel miRNAs (novel-mir3, novel-mir14, novel-mir15, novel-mir125, novel-mir140 and novel-mir321) were suppressed by higher temperature in the long photoperiod but did not fulfil the *p*-value cut off level in short-photoperiod conditions. Three known and four novel miRNAs were induced by higher temperature. Among these, miR827, novel-mir97 and novel-mir200 were induced specifically in the short photoperiod; miR8125 and novel-mir250 were stimulated only in the long photoperiod, while miR399 and novel-mir55 were significantly up-regulated in both conditions. In low temperatures, no miRNA expression was affected by the photoperiod. However, in high temperatures, two known miRNAs (miR399 and miR827) and three novel miRNAs (novel-mir170, novel-mir173 and novel-mir206) were suppressed by long-day conditions. Interestingly, one novel miRNA (novel-mir51) was down-regulated by both high temperature and long photoperiod considering the fold-changes. However, neither of them fulfilled the *p*-value < 10^− 3^ cut off level. When comparing the high-temperature, long-photoperiod to low-temperature, short-photoperiod conditions, both the fold-change and the *p*-value were improved to being past the cut off level. When comparing the high-temperature, short-day condition (as often happens in natural light greenhouses in the winter season) to the low-temperature, long-day condition (as often happens in low latitude and high elevation regions), the miRNA expression resembled that of the high vs. low temperature in the short-day condition. Comparison of the high-temperature, long-day condition with the low-temperature, short-day condition resembled the summer vs. winter season, showing similar miRNA expression patterns to high vs. low temperature in the long-day condition.

### Target prediction by degradome analysis

Using the psRobot [45] miRNA target prediction tool, 2027 and 6423 targets were assigned to the 66 known and 184 novel miRNAs (Additional file 1: Tables S6 and S7). Using another tool, TargetFinder [46], we predicted 2775 targets for known miRNAs and 8291 targets for novel miRNAs (Additional file 1: Tables S8 and S9). Assignments of 1007 of the known miRNA targets and 3604 of the novel miRNA targets were the same between the two methods (Additional file 1: Tables S10 and S11).

**Fig. 2:**
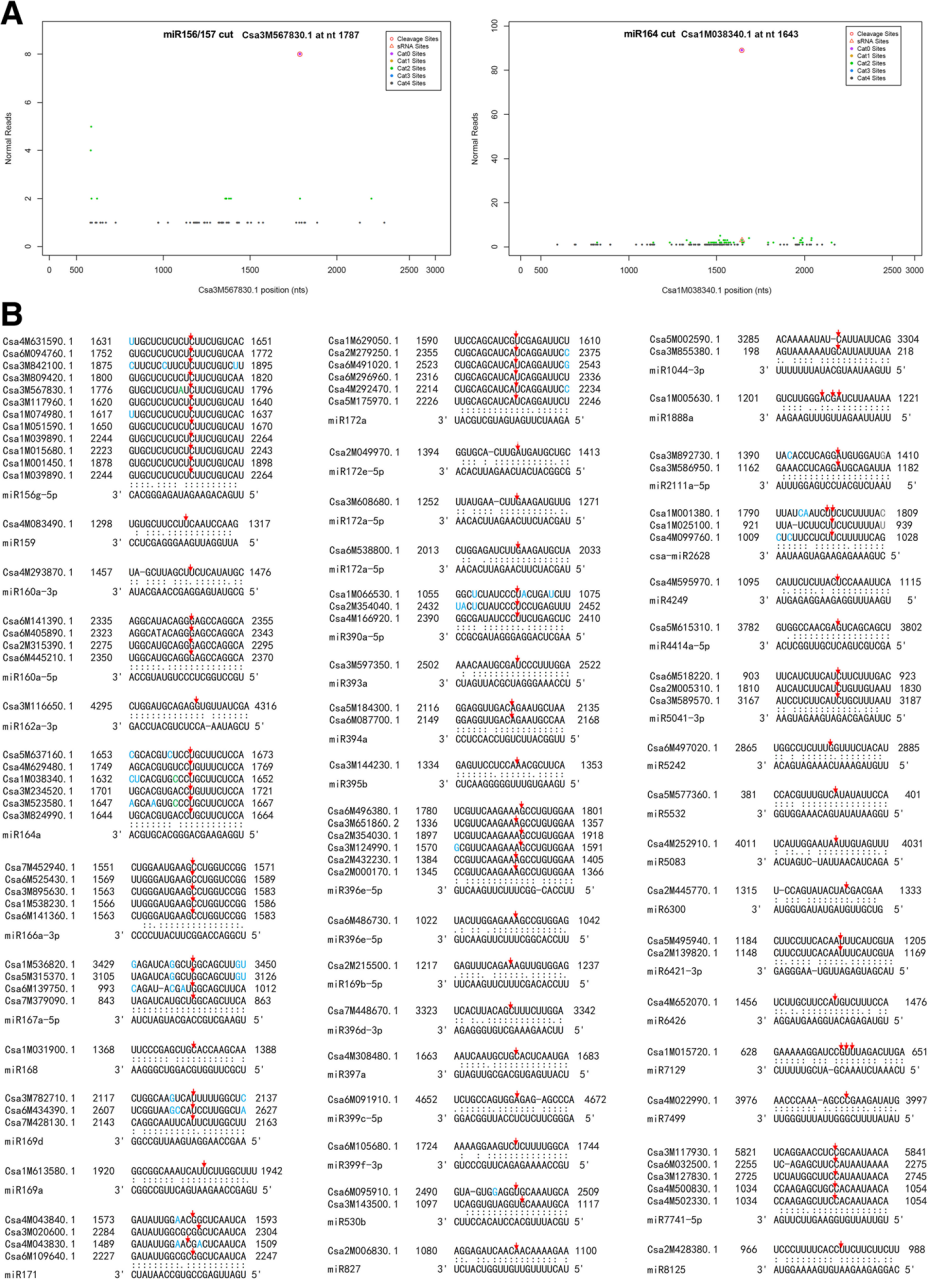
Degradome proven targets of known miRNAs. **a** Cleavage site plots of two typical targets. **b** Pairing of the known miRNAs with the mRNA target sites. The red arrows indicate the cut sites, the differential bases within a group are marked in blue

**Fig. 3:**
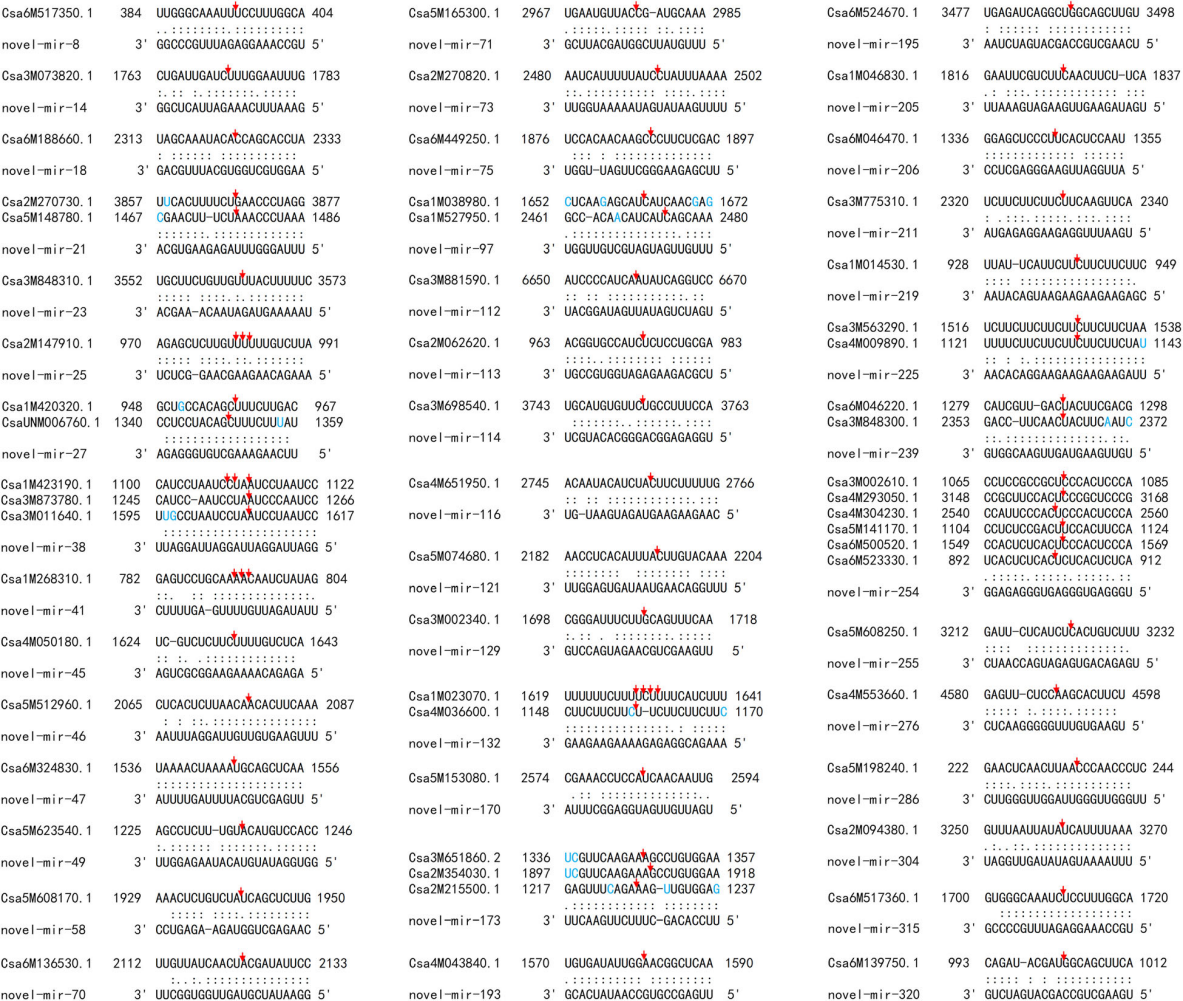
Pairing of the novel miRNAs (except novel-mir153) with the mRNA target sites. The red arrows indicate the cut sites, the differential bases within a group are marked in blue

**Fig. 4:**
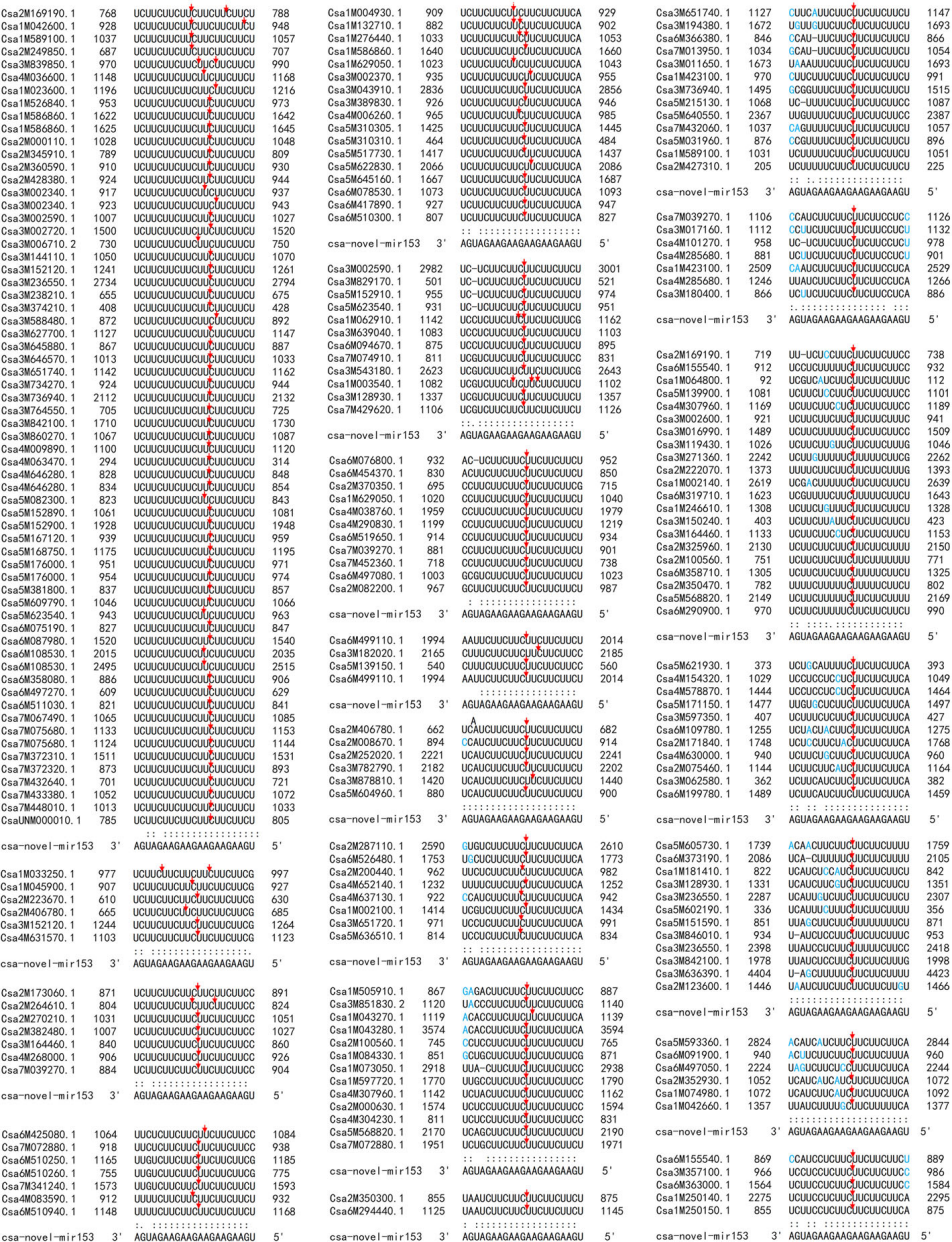
Pairing of the novel-mir153 with its mRNA target sites. The red arrows indicate the cut sites, the differential bases within a group are marked in blue

In plants, miRNAs function mainly by degrading their target mRNAs. Thus, we performed a degradome sequencing analysis to validate the miRNA targets. A total of 17.05 M clean tags were generated from the mixed samples of cucumber shoot tips cultivated in the four temperature-photoperiod conditions. Among them, 12.34 M (72.38%) tags were mapped to the reference cucumber (Chinese Long) genome (*Cucumis sativus* L. var. *sativus* cv. 9930 _v20) [47]. A total of 7.70 M (45.13%) tags were mapped to cDNA sense chains and were used for target prediction. A total of 349 mRNAs (394 target sites) were identified as miRNA targets, including 102 genes targeted by 37 known miRNA families and 256 genes targeted by 45 novel miRNAs. Amongst these, novel-mir153 had 232 targets on 201 genes. The cut sites are shown in (Figs. 2, 3 and 4) and the annotation of the targets are list in (Additional file 1: Table S12). A large proportion of miRNAs, including 31 known and 137 novel, had no targets detected, possibly due to the target mRNAs being expressed at low levels that the cut ends were not detected by our pipeline.

**Fig. 5:**
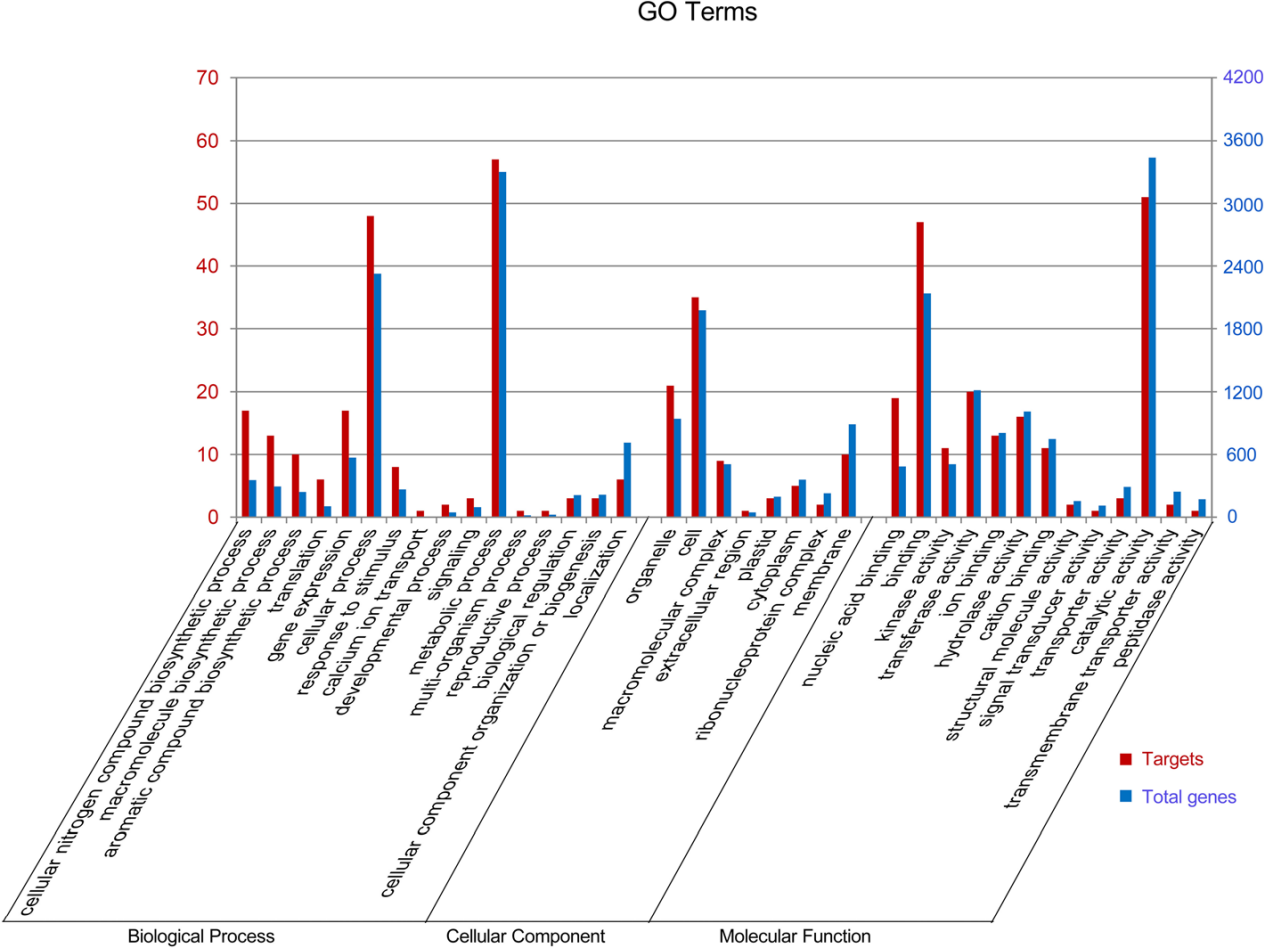
GO annotation of targets of miRNAs

The targets were classified by gene ontology (GO) annotation, with the top two terms being “metabolic process” and “cellular process” in the “biological process” cluster. “Cell” and “organelle” were the two most common “cellular component” targets. “Binding” and “catalytic activity” were the two most common terms in the “molecular function” cluster. The “cellular nitrogen compound biosynthetic process”, “macromolecule biosynthetic process”, “aromatic compound biosynthetic process”, “translation”, “gene expression”, “nucleic acid binding” and “binding” were the over-represented GO terms for the targets (Fig. 5, Additional file 1: Table S13). These targets were further categorized by KEGG, and the “Plant hormone signal transduction” pathway was the only significantly over-represented pathway (Additional file 1: Table S14). Among the 629 genes of this pathway, 27 were predicted as targets of miRNAs in cucumber shoot tips.

### Target expression profiles based on transcriptome sequencing

**Fig. 6:**
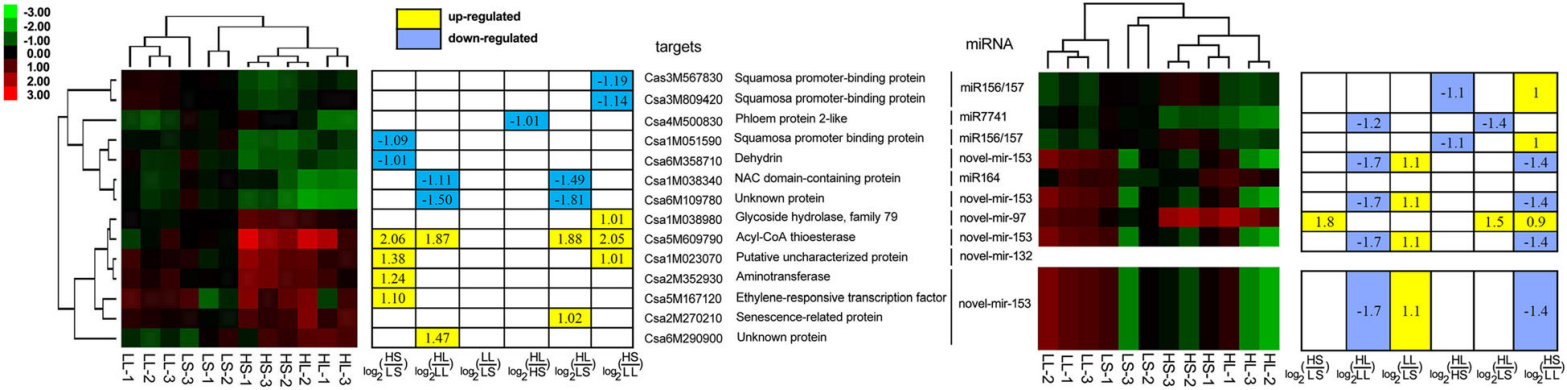
Heat maps of differentially expressed targets and their miRNAs

To profile the expression of the targets, a transcriptome sequencing project was performed using the same samples as those used for miRNA sequencing. A total of 25.35–27.60 M clean reads were generated from each of the 12 samples (three biological replicates for four treatments). For each sample, 19,045–19,242 genes were expressed (Additional file 1: Table S15). A total of 544 genes were differentially expressed between at least one pair of the six comparisons. The expression levels of the target genes were extracted from the transcriptome data. Amongst these, 14 targets of six miRNAs were differentially expressed among at least one pair of comparison. There were seven targets (one target of novel-mir97, one target of novel-mir132 and five targets of novel-mir153) were up-regulated and the six targets (three targets of miR156/157, one target of miR164 and two targets of novel-mir153) were down-regulated by high temperature, respectively. One gene targeted by miR7741 were down-regulated by long-day condition (Fig. 6, Additional file 1: Table S16). Amongst, novel-mir153 targeted to seven genes, which composed 50% of the total differentially expressed targets. However, the expression of novel-mir153 among the treatments displayed more than two-fold changes but did not reach the *p*-value< 10^− 3^ cut-off level. The accumulation of miR164 and novel-mir132 showed no significant different. Only three miRNAs, including miR156/157, miR7741, and cas-novel-mir97, were differentially expressed simultaneously with their targets (Additional file 1: Table S16).

### miRNA-target modulating the adaptation to temperature-photoperiod changes

**Fig. 7:**
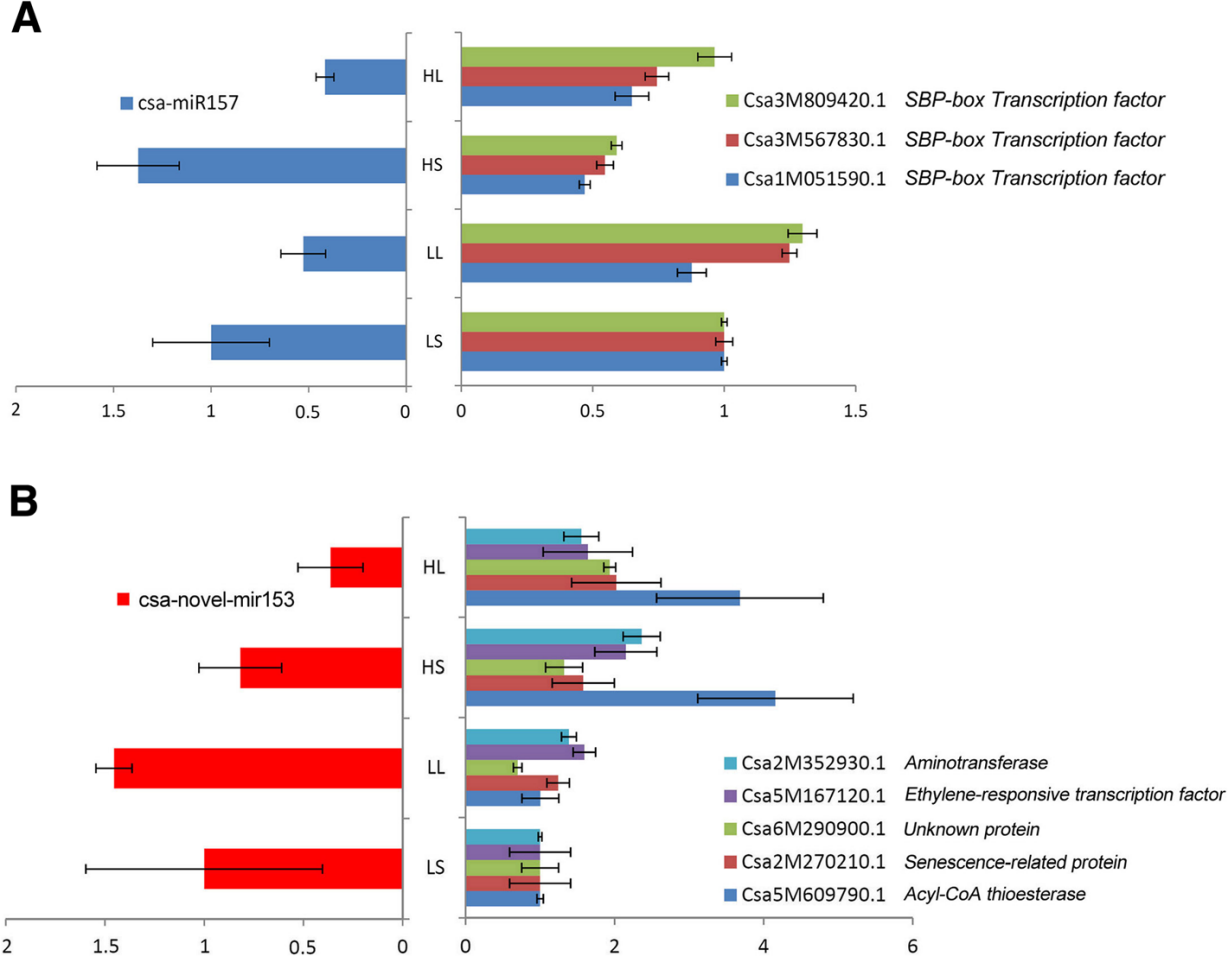
Negative correlation pattern of miRNAs and targets

The roles of the miRNA-target network in modulating the plant adaptation to temperature-photoperiod changes were investigated based on the functional annotations of the expressional affected miRNAs and targets. Of the 24 temperature and photoperiod affected miRNAs, 12 have 32 targets detected by degradome analysis (Additional file 1: Table S17). Five out of the 32 targets were differentially expressed (Additional file 1: Table S17). However, only miR156/157 and its three *SBP-box* targets have shown negative correlated expression pattern (Fig. 7a). MiR156/157 is well studied and negatively controls plant juvenile to adult phase changes, flowering, and other developmental processes via *SBP-box* targets [29, 48, 49]. By down-regulation of miR156/157, the long-day condition promoted the juvenile to adult phase changing, flower initiation and other developmental and metabolic processes in cucumber.

Though did not achieve the significantly different cut-off level, the expression of novel-mir153 among the treatments displayed negative correlation pattern with its five high-temperature induced targets (Fig. 7b). These targets include an aminotransferase, an ethylene-responsive transcription factor, a senescence-related protein and an acyl-CoA thioesterase coding gene. As ethylene is the central regulator signal for flower sex development in cucumber, the novel-mir153 has the potential to play roles in sex ratio regulation.

### A newly evolved novel miRNA targets a large number of genes

**Fig. 8:**
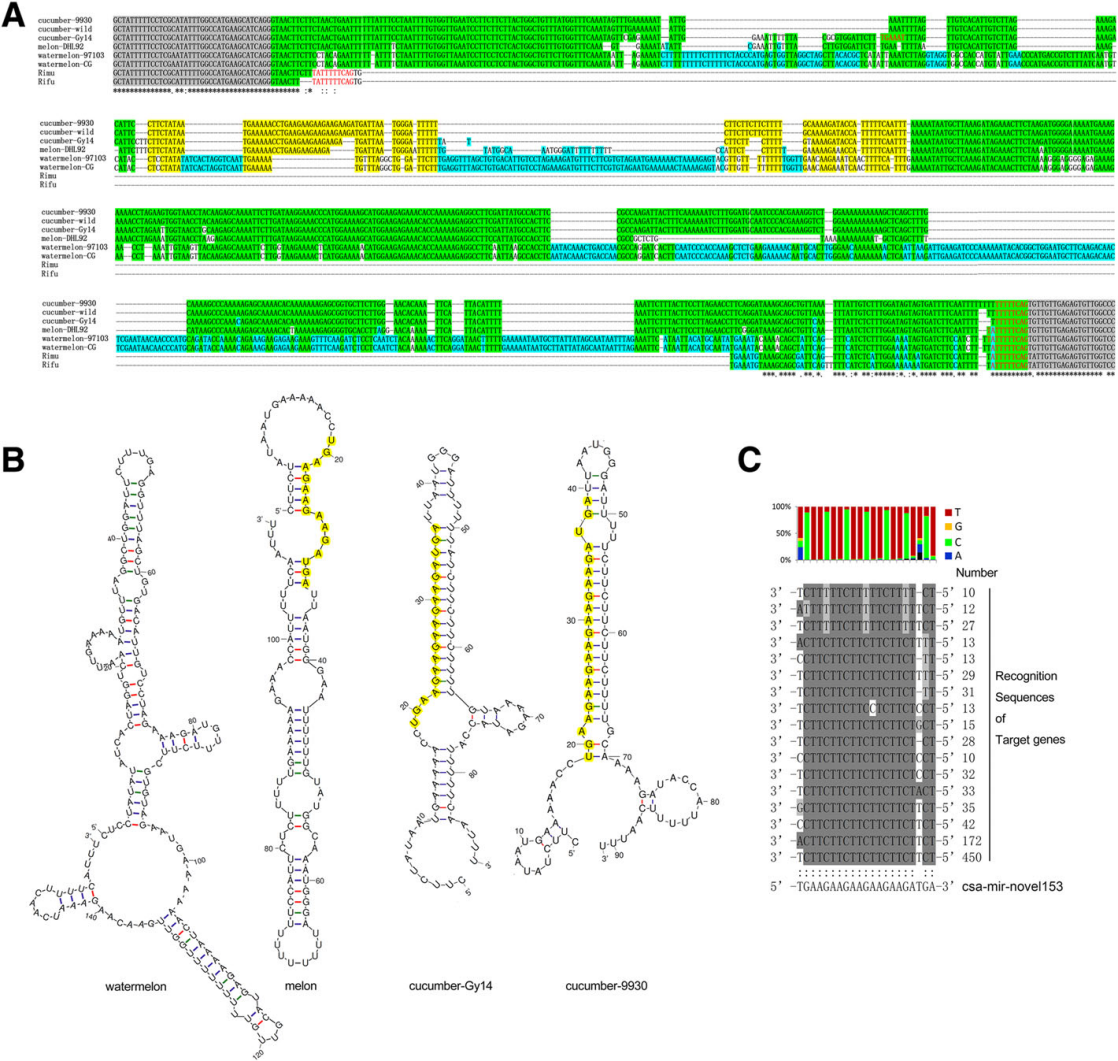
Evolution of novel-mir153 in cucumber. **a** alignment of novel-mir153 harbouring sequences of three cucumber genotypes and the homologous sequences in melon, watermelons and pumpkins. Grey shading shows the exons, the multicolour shading shows the intron, the green shading shows the conserved sequences, and the yellow shading shows the novel-mir153 hairpin sequences. The blue shading shows the watermelon retained sequences. **b** secondary structures of the novel-mir153 hairpin and its homologs in watermelon, melon and Gy14 cucumber. The yellow shading shows the mature RNA and its homologs. **c** alignment of novel-mir153 and its targets

Noticeably, a novel miRNA (novel-mir153) targeted 2087 mRNA targets, predicted by the intersection of the psRobot and TargetFinder tools, which accounted for 45.26% of the total targets in this plant. Amongst, 232 cut sites were proved by degradome analysis, which accounted for 58.88% of the total degradome results. This is the largest sum of genes targeted by a single miRNA in plants. By BLAST on NCBI, we did not find a similar hairpin structure sequence from other organisms. When this novel-mir153 precursor was traced back to the cucumber (Chinese Long 9930) genome, we noticed that it was generated from the third intron of the *alcohol dehydrogenase-like 6* gene (Csa1G044860). We then retrieved its homologue genes from seven other cucurbit genomes, including two cucumbers (Gy14 and PI183967), melon (DHL92), two watermelons (97,103 and Charleston Gray), *Cucurbita maxima* (Rimu) and *Cucurbita moschata* (Rifu) [50]. The coding sequences were highly conserved between these plants (Additional file 2: Figure S2). However, the intron regions have diverged between these species. The 3′-end part of the third intron was conserved between these five species, indicating that these introns were generated from a common ancestor. The direct repeat sequence at the borders indicates the intron was probably derived from transposable element insertion. The 5′-end of the third introns was lost in pumpkins or gained by the progenitor of cucumber, melon and watermelon after divergence from pumpkins. After this, two regions highly diverged between *Cucumis* and *Citrullus*. One of them evolved the novel-mir153 in cucumber (9930 and wild) by at least three steps (Fig. 8a and b). The secondary structure of the precursor of novel-mir153 showed perfect hairpin folding (Fig. 8b). This novel-miRNA is very young, evolved from the ancestors of melon and cucumber to cucumber cultivar Gy14 and then to cucumber cultivar Chinese long by two rounds of “AGA” duplications. The novel-mir153 and its targets matches are shown in Figs. 4 and 8c, perfectly meeting the criterion for regulation.

We checked the functions of these targeted genes, 182 out of the 201 genes were functionally annotated. These targets covered quite wide biological functions, including RNA transcription, protein translation, protein, lipid and carbohydrate metabolic, growth regulation, environmental response, transporters, etc. (Additional file 1: Table S12). It is worth mentioning that 23 transcription factors, nine kinase and seven cyclin-like genes were putative targets of novel-mir153, indicating this novel miRNA plays an important role in cucumber development.

## Discussion

Three previous studies by Ling et al., (2017); Mao et al., (2012) and Martinez et al., (2011) have identified 25, 29 and 31 known and 7, 2 and 49 novel families of miRNAs in cucumber plants (leaf, root and stem tissues) [42–44]. Among these previously identified miRNAs, 27 families of known miRNAs were also detected in our present study. The other eight known miRNA families (miR170, miR319, miR858, miR894, miR1030, miR1515, miR2911 and miR2916) identified in these previous reports were not detected in our study. However, our present study detected 41 new known miRNA families that have never been identified in previous reports (Table 1). These differences present the differential tissue patterns of miRNAs between the leaf, root, and stem in the previous reports and the shoot apex in our present study.

The expressional profiling showed that the miRNAs were primarily affected by temperature rather than by photoperiod. The temperature had an epistatic effect on photoperiod (Fig. 1). When cultivated in low temperature, the change of photoperiod did not affect the expression levels of miRNAs in these cucumber shoot tips. However, when temperature rose above a suitable level, the photoperiod significantly influenced the expression of many miRNAs. These results give guidance for cucumber production, especially for greenhouse-based cultivation, in that temperature has a greater impact than photoperiod and merits greater attention. When cucumbers are growing in a greenhouse during the winter season, if the temperature is not high enough, the extension of the photoperiod will be insufficient. Only when the temperature are high enough will a prolonged photoperiod stimulate flowering and other developmental processes. However, cucumber has many ecotypes. The “Chinese long (9930)” used in this study is a cultivar successfully used in greenhouse cultivation in winter and early spring seasons. The above findings should be cautiously used if extended to other ecotype cucumbers such as the “Xishuangbanna” variety.

The functional annotations of the differentially expressed miRNAs provide some answers for the performance of cucumber within differential temperature-photoperiod environments (Additional file 1: Table S18**,** Additional file 2: Figure S3). For example, down-regulation of miR7741 and seven other miRNAs releases the activity of targets involved in transcription initiation, elongation, mRNA processing, degradation, and protein translation in high temperature. This can explain the fact that these plants are more vigorous in higher temperatures. Under miRNA regulation, the expression or translation of proton-dependent transporter genes and vacuolar transport genes were biased towards high or low temperatures, respectively. This can contribute to the phenomena of mineral element absorbance and transport being more efficient in high temperatures, while the carbohydrate and alkaloid storage in vacuoles is more efficient in low temperatures. It is well known that low temperatures can cause fungal diseases in cucumber. The suppression of the resistance and defence related genes via miRNAs can partially explain this phenomenon. However, due to the limited sequencing depth, a lot of predicted targets were not proven in the degradome analysis. Therefore, deeper degradome sequencing is needed to prove these functional deductions.

One of the important outputs of the temperature-photoperiod influence is the flower sex ratio changes. DNA methylation has been proven to play a role in this process [11]. DNA methylation achieves its functions by transcriptional regulation of genes. miRNA is a more rapid and direct regulator of gene expression. In this study, miR156/157 differentially expressed and regulated the *SBP-box* transcription factors. *SBP-box* genes regulate flowering time, male fertility and gynoecium patterning in Arabidopsis [48, 49] but did not play a strong role in sex determination, according to the studies that were carried out using hermaphrodite flowering plants. Thus, we cannot exclude the possibility of these SBP-box transcription factors playing roles in flower sex ratio regulation in cucumber. Beside the significant differentially expressed miRNAs, those miRNAs such as novel-mir153 target *Ethylene-responsive transcription factors* whose expression was significantly affected by temperature and photoperiod. As ethylene is the central regulator signal for flower sex development in cucumber, these genes and the corresponding miRNAs have the potential to play roles in sex ratio regulation.

There are three models that describe miRNA origin and evolution: origination from a random formation of hairpins in non-coding sections such as introns or intergenic regions; origination from inverted duplications of protein-coding sequences; and origination from the miniature inverted-repeat transposable elements [51, 52]. In this study, we found a solid example of a new miRNA (novel-mir153) that originated from an intron. The comparison of the genes from pumpkins, watermelons, melons and cucumbers displayed the step-by-step evolution of this miRNA. Because novel-mir153 is a quite newly evolved miRNA and absent in other plants, it is not an indispensable element for the plant life cycle. However, it does target a surprisingly large number of target genes. The detailed function of novel-mir153 and its contribution to cucumber evolution is worthy further investigation.

## Materials and methods

### Plant materials and RNA extraction

The cucumber inbred line 9930 was used in this study. Seedlings were grown in artificial climate chambers with conditions set as follows: 16 h light in 28 °C /8 h dark in 25 °C (high temperature, long day, HL), 8 h light in 28 °C /16 h dark in 25 °C (high temperature, short day, HS), 16 h light in 20 °C /8 h dark in 15 °C (low temperature, long day, LL), and 8 h light in 20 °C /16 h dark in 15 °C (low temperature, short day, LS). A 70% humidity was used for all of these plants. When four true-leaves were unfolded, shoot apical tissues (1 mm) were dissected under a microscope and snap-frozen in liquid nitrogen and kept at − 80 °C for further use. In each experiment, more than 500 shoot apices and three biological replicates were used. Total RNA was extracted using the TRIzol reagent (Invitrogen, USA). DNase (Promega, USA) was used to remove potential DNA contamination.

### Small RNA library construction and sequencing

The RNA samples were quantified and equalized. A total of 30 μg of RNA was resolved on denatured polyacrylamide gels. Gel fragments with the size range of 18–30 nt were excised and recovered. These small RNAs were ligated with 5′ and 3’RNA adapters using T4 RNA ligase. The adapter-ligated small RNAs were subsequently transcribed into cDNA by Super-Script II Reverse Transcriptase (Invitrogen) and amplified using primers specific for the ends of the adapters. The amplified cDNA products were purified and finally sequenced using Illumina sequencing technology (BGI, Shenzhen, China).

### Identification of known and novel miRNAs

The adapter sequences, impurities, and sequences with less than 18 nt or more than 30 nt were filtered out from the raw sequence reads. For miRNA identification, the total reads were converted to non-redundant unique tags to filter out duplicates. The unique tags were aligned to GenBank, Rfam and the cucumber genome [47] and therefore annotated as rRNA, miRNA, snRNA, snoRNA, tRNA, repeat, exon sense, exon antisense, intron sense, intron antisense, and unannotated sequences. The putative miRNA tags, unannotated reads and those derived from the intron regions and exon antisense regions were used for miRNA mining based on the criteria as follow: hairpin pre-miRNAs that can fold into secondary structures and mature miRNAs that were present in one arm of the hairpin precursors; the 5-p and 3-p mature miRNAs present 2-nucleotide 3′ overhangs; hairpin precursors lacking large internal loops or bulges; the secondary structures of the hairpins were steady, with a free energy of hybridization lower than or equal to − 18 kcal/mol; and the copy number of mature miRNAs with predicted hairpins should be greater than 5 in the alignment result. The candidates were first aligned to all known plant miRNAs in miRBase 21.0 allowing a two-base mismatch and a two-nucleotide overhang. The matched miRNAs were termed as known miRNAs and named after their best-match families. The members within the same miRNA family were alphabetical labelled according to the genome location of their precursors. The rest of the miRNAs that could not be incorporated into known families were termed as novel miRNAs.

The expression of miRNAs was produced by summing the total count of tags with no more than 3 mismatches on the 5′ and 3′ ends and no mismatches in the middle from the alignment result. The differentially expressed miRNAs were calculated with the following procedures. First, the expression of miRNAs was normalized to obtain the expression of transcript per million (TPM). The normalization formula was as follows: Normalized expression = Actual miRNA count/Total count of clean reads× 1,000,000. Second, the fold-change and *P*-value were calculated from the normalized expressions.

### Degradome library construction and target identification

Total RNA was extracted from the same samples as those used for sRNA sequencing. Approximately 200 μg of total RNA was polyadenylated using the Oligotex mRNA mini kit (Qiagen). A 5′ RNA adapter was added to the cleavage products (which possessed a free 5′-monophosphate at their 3′ termini) using the T4 RNA ligase (Takara). Then, the ligated products were purified using the Oligotex mRNA mini kit (Qiagen) for reverse transcription to generate the first strand of cDNA using an oligo dT primer via SuperScript II RT (Invitrogen). After the cDNA library was amplified for 6 cycles (94 °C for 30 s, 60 °C for 20 s, and 72 °C for 3 min) using Phusion Taq (NEB), the PCR products were digested with restriction enzymes and further ligated to double-stranded DNA adapters. The ligated products were subjected to PCR amplification and gel purification and finally used for high-throughput sequencing with the Illumina HiSeq 2000.

Low-quality sequences and adapters were removed, and the unique sequence signatures were aligned to the cucumber (9930) genome [47] using SOAP software [53]. CleaveLand was used to detect potentially cleaved targets [54]. The tags mapped to cDNA sense strands were adopted to predict cleavage sites. The miRNA-mRNA pairs and *p*-values were analysed using PAREsnip, *p*-values less than 0.05 were used for the t-plot [55]. All alignments with scores not exceeding 4 and possessing the 5′ end of the degradome sequence coincident with the tenth and eleventh nucleotides of the miRNA complementary were retained. The targets were GO annotated using Blast2GO [56].

### Target gene profiling

Total RNAs (10 μg) were subjected to poly-A selection, fragmentation, random priming and cDNA synthesis with the Illumina Gene Expression Sample Prep kit (CA, USA). The cDNA fragments were end repaired, ligated to adapters and then enriched by PCR. The fragments were purified with 6% TBE PAGE gel electrophoresis. After denaturation, the single-chain fragments were fixed onto the Solexa Sequencing Chip (Flowcell) and consequently grown into single-molecule cluster sequencing templates through in situ amplification [57]. Double-end pyrosequencing was performed on the Illumina HiSeq 2000 with the read lengths of 90 bp for each end. The clean reads were aligned to cucumber reference genes [47]. Gene expression levels were calculated using the FPKM. Statistical comparison between treatments was performed using the data from three biological replicates. Unigenes were considered to be differentially expressed when the mean FPKM between treatments displayed a more than two-fold change with a probability of more than 0.8. The expression profiles of the target genes were extracted from the transcriptome data.

## Additional files

Additional file 1: **Table S1.** summary of sRNA sequencing data. **Table S2.** summary of known miRNAs. **Table S3.** summary of novel miRNAs. **Table S4.** expression levels of known miRNAs in cucumber shoot tips grown in four temperature-photoperiod conditions. **Table S5.** expression levels of novel miRNAs in cucumber shoot tips grown in four temperature-photoperiod conditions. **Table S6.** targets of known miRNAs predicted by psRobot. **Table S7.** targets of novel miRNAs predicted by psRobot. **Table S8.** targets of known miRNAs predicted by TargetFinder. **Table S9.** targets of novel miRNAs predicted by TargetFinder. **Table S10.** targets of known miRNAs predicted by both psRobot and TargetFinder (intersection). **Table S11.** targets of novel miRNAs predicted by both psRobot and TargetFinder (intersection). **Table S12.** targets identified by degradome sequencing. **Table S13.** GO term enrichment of targets. **Table S14.** KEGG classification of miRNA targets. **Table S15.** statistics of transcriptome sequencing for each sample. **Table S16.** differentially expressed degradome confirmed targets. **Table S17.** targets (degradome proven) of the temperature and photoperiod affected miRNAs. **Table S18.** functional annotations (computer predicted targets) of the temperature and photoperiod affected miRNAs. (XLSX 1042 kb)

Additional file 2: **Figure S1.** Length distribution of small RNAs in cucumber shoot tips. **Figure S2.** Alignment of exons of *alcohol dehydrogenase-like 6* genes **Figure S3.** Schematic diagram of the miRNA-target network modulating the plant adaptation to temperature changes. (DOCX 590 kb)
