# Supplementary material for: MicroRNAs and their targets in cucumber shoot apices in response to temperature and photoperiod
Source: BMC Genomics. 2018 Nov 15;19:819. doi: 10.1186/s12864-018-5204-x (PMC6238408; doi:10.1186/s12864-018-5204-x)
Supplement: Supplementary file 2 — Figure S1. Length distribution of small RNAs in cucumber shoot tips. Figure S2. Alignment of exons of alcohol dehydrogenase-like 6 genes Figure S3. Schematic diagram of the miRNA-target network modulating the plant adaptation to temperature changes. (DOCX 590 kb) [file 12864_2018_5204_MOESM2_ESM.docx]

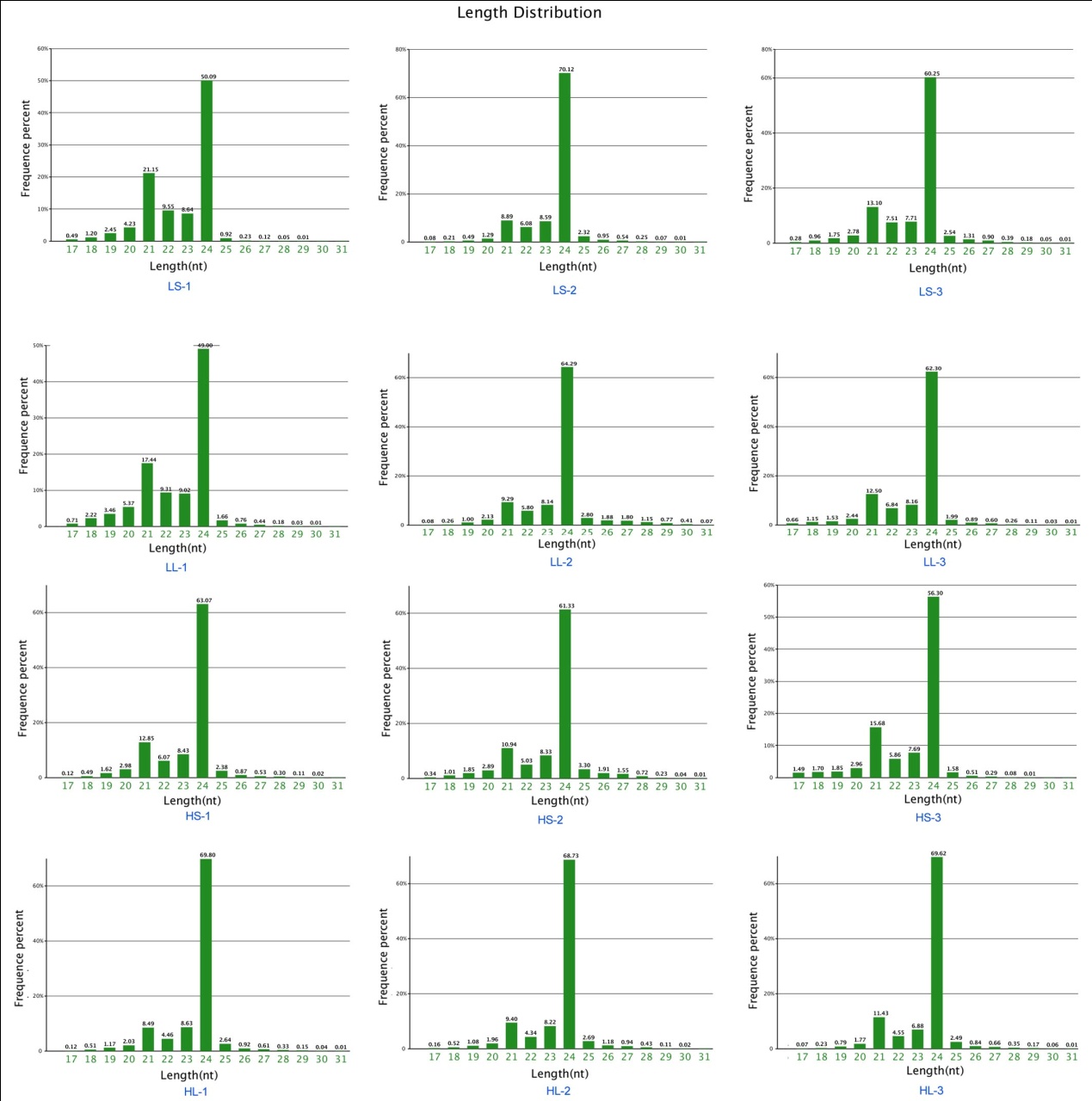


**Figure S1 Length distribution of small RNAs in cucumber shoot tips**

Cucsa.097760-r ------------------------------------------------------------

Cla008358-r ------------------------------------------------------------

MELO3C002189-r ------------------------------------------------------------

CSPI01G07780-r ------------------------------------------------------------

CmaCh13G009390-r CGCCCCCCTCCATCTCCACCAACCGCACCTACCACCAAAGGCGAATGATAGAAACTCGCA

Cucsa.097760-r ------------------------------------------------------------

Cla008358-r ------------------------------------------------------------

MELO3C002189-r ------------------------------------------------------------

CSPI01G07780-r ------------------------------------------------------------

CmaCh13G009390-r GCTCCTTCGCCTGTTCAAGGCGACTCAATACAATTCTTCCCAGACTCCGCTGGAAGAATT

Cucsa.097760-r ---------------------------------ATGTCCTCCTCGTCTTCTTCTTTCCCG

Cla008358-r ------------------------------------------ATGTCGTCTTCTTCCCCG

MELO3C002189-r ------------------------------ATGTCCTCCTCCTCGTCTTCTTCTTTCCCG

CSPI01G07780-r ---------------------------------ATGTCCTCCTCGTCTTCTTCTTTCCCG

CmaCh13G009390-r CCCTCCGAATCAGTATTTTTCTTGCGCTTCAATCTCACCGGGATGTCGTCTTCTTCCCCT

: *** ******* ***

Cucsa.097760-r GCGGTTCTTACTTGCAAAGCCGCGGTCGCTTGGGGACCTGGACAACCATTGGTGATTGAG

Cla008358-r GCGGTTCTTACTTGCAAAGCCGCGGTGGCTTGGGGACCTGGAGAGCCATTGGTGATTGAG

MELO3C002189-r GCGGTTCTTACTTGCAAAGCCGCGGTCGCTTGGGGACCTGGACAACCATTGGTGATTGAA

CSPI01G07780-r GCGGTTCTTACTTGCAAAGCCGCGGTCGCTTGGGGACCTGGACAACCATTGGTGATTGAA

CmaCh13G009390-r GCGGTTCTTACTTGCAAAGCCGCGGTCGCTTGGGGACCTGGAGAGCCATTGGTGATTGAG

************************** *************** *.**************.

Cucsa.097760-r GAGGTGGAAGTCAGTCCTCCTCAACCTATGGAAATCAGAGTTAAAGTTGTTTCTACTTCT

Cla008358-r GAGGTGCAAGTGAGTCCTCCTCAACCTATGGAAATCAGAATTAAAGTTGTTTGCACTTCT

MELO3C002189-r GAGGTGGAAGTCAGTCCTCCTCAACCCATGGAAATCAGAATTAAAGTTGTTTCTACTTCT

CSPI01G07780-r GAGGTGGAAGTCAGTCCTCCTCAACCTATGGAAATCAGAGTTAAAGTTGTTTCTACTTCT

CmaCh13G009390-r GAGGTGCAAGTGAGTCCTCCTCAACCTATGGAGATCAGAATTAAAGTTGTCTGCACTTCT

****** **** ************** *****.******.********** * ******

Cucsa.097760-r TTGTGTCGCAGCGATCTCTCTGCTTGGGAAACTCAGGCTATTTTTCCTCGCATATTTGGC

Cla008358-r TTGTGTCGCAGCGATGTTTCTGCTTGGGAAACTCAGGCTATTTTTCCTCGAATATTTGGC

MELO3C002189-r TTGTGTCGCAGCGATCTCTCTGCTTGGGAAACTCAGGCTATTTTTCCTCGCATATTTGGC

CSPI01G07780-r TTGTGTCGCAGCGATCTCTCTGCTTGGGAAACTCAGGCTATTTTTCCTCGCATATTTGGC

CmaCh13G009390-r TTGTGTCGCAGTGATTTCTCTGCGTGGGAAACTCAGGCTATTTTTCCTCGCATTTTTGGC

*********** *** * ***** **************************.**:******

Cucsa.097760-r CATGAAGCATCAGGTGTTGTTGAGAGTGTTGGCCCTGGAGTGACTGAATTCAGTGAAGGT

Cla008358-r CATGAAGCATCAGGTGTTGTTGAGAGTGTTGGTCCTGGAGTGACTGAATTCAGTGAAGGT

MELO3C002189-r CATGAAGCATCAGGTGTTGTTGAGAGTGTTGGCCCTGGAGTGACTGAATTCAGTGAAGGT

CSPI01G07780-r CATGAAGCATCAGGTGTTGTTGAGAGTGTTGGCCCTGGAGTGACTGAATTCAGTGAAGGT

CmaCh13G009390-r CATGAAGCATCAGGTATTGTTGAGAGTGTTGGTCCTGGAGTGACTGAATTCAGTGAAGGT

***************.**************** ***************************

Cucsa.097760-r GACCATGTGCTTACACTATTCACTGGAGAATGCAAGACATGTAGACATTGCACATCAGGT

Cla008358-r GACCATGTGCTTACACTATTCACTGGAGAATGCAAGACATGTAGGCATTGCACATCCGGT

MELO3C002189-r GACCATGTGCTCACACTATTCACTGGAGAATGCAAGACATGTAGACATTGCACATCAGGT

CSPI01G07780-r GACCATGTACTTACACTATTCACTGGAGAATGCAAGACATGTAGACATTGCACATCAGGT

CmaCh13G009390-r GATCACGTGCTTACACTATTCACTGGAGAATGCAAAACATGTAGGCAGTGCACATCAGGT

** ** **.** ***********************.********.** ********.***

Cucsa.097760-r AAAAGCAACATGTGCCAAGTTTTGGGACTAGAGAGGAGAGGTGTAATGCATAGTGATCAG

Cla008358-r AAAAGCAACATGTGCCAAGTTTTGGGACTAGAGAGGAGAGGTGTAATGCATAGTGATCAA

MELO3C002189-r AAAAGCAACATGTGCCAAGTTTTGGGACTAGAGAGGAGAGGTGTAATGCACAGTGATCAG

CSPI01G07780-r AAAAGCAACATGTGCCAAGTTTTGGGACTAGAGAGGAGAGGTGTAATGCATAGTGATCAG

CmaCh13G009390-r AAAAGCAATATGTGCCAAGTTTTGGGACTAGAGAGGAGAGGTGTAATGCATAGTGATCAG

******** ***************************************** ********.

Cucsa.097760-r AAGACCCGCTTCTCTATCAAAGGCAAACCAATTTATCATTATTGTGCGGTTTCAAGTTTC

Cla008358-r AAGACCCGCTTCTCTATCAAAGGCAAACCAATTTATCATTATTGTGCGGTTTCAAGTTTC

MELO3C002189-r AAGACCCGCTTCTCTATCAAAGGCAAACCAATTTATCATTATTGTGCGGTTTCAAGTTTC

CSPI01G07780-r AAGACCCGCTTCTCTATCAAAGGCAAACCAATTTATCATTATTGTGCGGTTTCAAGTTTC

CmaCh13G009390-r AAGACCCGATTCTCTATCAAAGACAAACCAGTTTATCATTATTGTGCGGTTTCAAGTTTC

********.*************.*******.*****************************

Cucsa.097760-r AGTGAATACACCGTGGTGCACTCAGGGTGTGCTGTCAAAGTCAGCTTGGCTGTACCTCTT

Cla008358-r AGCGAGTACACCGTGGTGCACTCAGGGTGTGCTGTCAAAGTCAACTTGGCTGTACCTCTT

MELO3C002189-r AGTGAATATACCGTGGTGCACTCAGGGTGTGCTGTCAAAGTCAGTTTGGCTGTACCTCTT

CSPI01G07780-r AGTGAATACACCGTGGTGCACTCAGGGTGTGCTGTCAAAGTCAGCTTGGCTGTACCTCTT

CmaCh13G009390-r AGTGAATATGCCGTGGTGCACTCAGGGTGTGCTGTCAAAGTCAGCTTGGCTGTACCTCTT

** **.** .*********************************. ***************

Cucsa.097760-r GAGAAAATATGTCTTTTAAGCTGCGGGGTGGCCGCAGGTTTGGGTGCCGCTTGGAATGTT

Cla008358-r GAGAAAATATGTCTTTTAAGCTGTGGGGTGGCCGCAGGTTTGGGTGCTGCTTGGAATGTC

MELO3C002189-r GAGAAAATATGTCTTTTAAGCTGCGGTGTGGCCGCAGGTTTGGGTGCCGCTTGGAATGTT

CSPI01G07780-r GAGAAAATATGTCTTTTAAGCTGCGGGGTGGCCGCAGGTTTGGGTGCCGCTTGGAATGTT

CmaCh13G009390-r GAGAAAATATGTCTTTTAAGTTGTGGAGTGGCTGCAGGGTTGGGTGCTGCTTGGAATGTC

******************** ** ** ***** ***** ******** ***********

Cucsa.097760-r GCTGATATATCTGAAGGTTCAACTGTGGTGATATATGGTCTGGGGACTGTAGGCCTATCT

Cla008358-r GCTGATATATCTGATGGTTCAACTGTGGTGATATATGGTCTCGGGACTGTAGGCCTATCT

MELO3C002189-r GCTGATATATCTAAAGGTTCAACTGTGGTGATATATGGTCTAGGGACTGTAGGCCTATCT

CSPI01G07780-r GCTGATATATCTGAAGGTTCAACTGTGGTGATATATGGTCTGGGGACTGTAGGCCTATCT

CmaCh13G009390-r GCTGATATATCTGAAGGTTCAACTGTGGTGATATATGGTCTTGGGACTGTAGGTCTATCT

************.*:************************** *********** ******

Cucsa.097760-r GTTGCACAAGGTGCCAAAGTTAGGGGTGCGTCGCAAATAATTGGTGTTGATATTAATCCT

Cla008358-r GTTGCACAAGGTGCCAAAGTTAGGGGTGCATCGCAAATAATTGGTGTTGATATTAACCCT

MELO3C002189-r GTTGCACAAGGTGCTAAAGTTAGGGGTGCGTCGCAAATAATTGGTGTTGATATTAATCCT

CSPI01G07780-r GTTGCACAAGGTGCCAAAGTTAAGGGTGCGTCGCAAATAATTGGTGTTGATATTAATCCT

CmaCh13G009390-r GTTGCACAAGGTGCCAAAGTTAGGGGTGCATCGCAAATAATTGGCGTTGATATTAATCCT

************** *******.******.************** *********** ***

Cucsa.097760-r GAAAAGAGTGAAATAGCTAAAACTTTTGGAATTACTCATTTTGTTAATCCGAAGGAATGC

Cla008358-r GAAAAGGGTGAAATAGCTAAAACTTTTGGAATTACCCATTTTGTTAACCCGAAGGAATGC

MELO3C002189-r GAAAAGAGCGAAATAGCCAAAACTTTTGGGGTTACTCATTTTGTTAACCCGAAGGAATGC

CSPI01G07780-r GAAAAGAGTGAAATAGCTAAAACTTTTGGAATTACTCATTTTGTTAATCCGAAGGAATGC

CmaCh13G009390-r GAAAAGGGTGAAATAGCCAAAACTTTTGGGATCACTCATTTTGTTAATCCGAAGGAGTGC

******.* ******** ***********..* ** *********** ********.***

Cucsa.097760-r AGTGAATCTATTCAACAGGTCATTAATCGAATTACCGATGGAGGAGCTGATTATGCATTT

Cla008358-r AATGAATCTATTCAACAGGTCATAAATCAAATTACTGAGGGAGGGGCTGATTATGCATTT

MELO3C002189-r AATGATTCTATTCAACAGGTCATTAATCGAATTACTGATGGAGGAGCTGATTATGCATTT

CSPI01G07780-r AGTGAATCTATTCAACAGGTCATTAATCGAATTACTGATGGAGGAGCTGATTATGCATTT

CmaCh13G009390-r AATGAATCCATTCAACAGGTCGTGAATCGAATTACTGATGGCGGGGCTGATTATGCATTT

*.***:** ************.* ****.****** ** **.**.***************

Cucsa.097760-r GAGTGTATAGGTGACACAGGAATGATTACTACTGCTTTGCAGTCATGTTGTCAAGGTTGG

Cla008358-r GAATGTATAGGTGACACAGGAATGATAACTACTGCTTTGCAGTCATGTTGTCAAGGTTGG

MELO3C002189-r GAATGTATAGGTGACACAGGAATGATTACTACTGCTTTGCAGTCATGTTGTCAAGGTTGG

CSPI01G07780-r GAGTGTATAGGTGACACAGGAATGATTACTACTGCTTTGCAGTCATGTTGTCAAGGTTGG

CmaCh13G009390-r GAATGTATAGGTGACACAGGAATGATAACTACTGCATTGCAGTCCTGTTGTCAAGGTTGG

**.***********************:********:********.***************

Cucsa.097760-r GGTCTGACTGTTACACTAGGTGTCCCAAAAGTGAATCCAGAATTAACGGCCCACTATGGA

Cla008358-r GGTCTGGCTGTTACACTAGGTGTCCCGAAAGTGAATCCAGAACTAACGGCCCACTATGGA

MELO3C002189-r GGTCTGACTGTAACACTAGGTGTCCCAAAAGTGAATCCAGAATTAACAGCCCACTATGGA

CSPI01G07780-r GGTCTGACTGTTACACTAGGTGTCCCAAAAGTGAATCCAGAATTAACGGCCCACTATGGA

CmaCh13G009390-r GGTTTGACTGTTACATTAGGTGTCCCAAAAGTGAATCCAGAACTAACAGCCCACTATGGA

*** **.****:*** **********.*************** ****.************

Cucsa.097760-r ATACTTCTTAGTGGAAGAACACTAAGAGGATCCCTTTTTGGCGGATGGAAACCAAAATCT

Cla008358-r ATATTTCTTAGTGGAAGAACATTAAAAGGATCCCTTTTTGGCGGATGGAAACCAAAATCT

MELO3C002189-r ATGTTTCTTAGTGGAAGAACATTAAAAGGATCCCTTTTTGGCGGATGGAAACCAAAATCT

CSPI01G07780-r ATACTTCTTAGTGGAAGAACACTAAGAGGATCCCTTTTTGGCGGATGGAAACCAAAATCT

CmaCh13G009390-r GTATTTCTTAGTGGGAGAACATTAAAAGGATCCCTTTTTGGCGGATGGAGACCAAAATCT

.*. **********.****** ***.***********************.**********

Cucsa.097760-r GATCTCCCCTCATTAGTAGACATGTATACCAAGAAGGAAATTCAAATTGACGAGTACATC

Cla008358-r GATCTCCCCTCATTAGTAGACATGTATACCAAGAAGGAAATTCAAATCGACGAGTACATC

MELO3C002189-r GATCTCCCCTCATTAGTAGACATGTATTCAAAGAAGGACATTCAAATTGATGAGTACATC

CSPI01G07780-r GATCTCCCCTCATTAGTAGACATGTATACCAAGAAGGAAATTCAAATTGACGAGTACATC

CmaCh13G009390-r GATCTCCCCTCATTGGTAGACATGTATACCAAGAAGGAAATTCAAATTGACGAGTACATC

**************.************:*.********.******** ** *********

Cucsa.097760-r ACACACAACATATCCTTTGAAGATATCAACCAAGCTTTCACTCTCATGAAGGAAGGGAAG

Cla008358-r ACTCATAACATATCCTTTGAAGATATCAACCAAGCTTTCAGTCTCATGAAGGAAGGGAAG

MELO3C002189-r ACACACAACATATCCTTTGAAGATATCAACCAAGCTTTCACTCTCATGAAGGAAGGGAAG

CSPI01G07780-r ACACACAACATATCCTTTGAAGATATCAACCAAGCTTTCACTCTCATGAAGGAAGGGAAG

CmaCh13G009390-r ACACACAACATATCCTTTGAAGATATCAACCAAGCTTTCAGTCTCATGAAGGAAGGGAAG

**:** ********************************** *******************

Cucsa.097760-r TGTTTACGTTGTGTTATTCACTTACCAGCATGA---------------------------

Cla008358-r TGTTTACGCTGTGTTATTCACTTACCAGCATGA---------------------------

MELO3C002189-r TGTTTACGTTGTGTTATTCACTTACCAGCATGATCTTGAATTAATTAAACCCATCTTGGA

CSPI01G07780-r TGTTTACGTTGTGTTATTCACTTACCAGCATGA---------------------------

CmaCh13G009390-r TGTTTACGTTGTGTTATTCACTTACCAGCATGATTTTATATTAATTAAATCCATCTTCGG

******** ************************

Cucsa.097760-r ------------------------------------------------------------

Cla008358-r ------------------------------------------------------------

MELO3C002189-r TGATTTTG----GTGAGTCAAATTCTTGGTTCTCCATGTCTGAATATAATTGGTGG--GA

CSPI01G07780-r ------------------------------------------------------------

CmaCh13G009390-r TGCTTTTTGCTAGTTGTTCAAATTCCTGGTTTTCTATTTCGTAATCTATTTGCTGGGAGT

Cucsa.097760-r ------------------------------------------------------------

Cla008358-r ------------------------------------------------------------

MELO3C002189-r GTTGATATTTGTACCATTCTTTAAATTGAGATAATTGAGAGCTTTATTATTATT------

CSPI01G07780-r ------------------------------------------------------------

CmaCh13G009390-r TCTGATATTTGTACGCTTCGTTGGATTGAGATATTTTGAGGTGCTTTTTTTGGGGGGTAA

Cucsa.097760-r ------------------------------------------------------------

Cla008358-r ------------------------------------------------------------

MELO3C002189-r ------------------------------------------------------------

CSPI01G07780-r ------------------------------------------------------------

CmaCh13G009390-r AGAATAACATTGTTACTTAGAATCAAGACTTAGGGCCTTTTGTTTTAAAATTCCTCGAGG

Cucsa.097760-r ------------------------------------------------------------

Cla008358-r ------------------------------------------------------------

MELO3C002189-r ------------------------------------------------------------

CSPI01G07780-r ------------------------------------------------------------

CmaCh13G009390-r AGCAGAGATATTTTGAAGCTTCCTGGGCATTAAATTTCATGAACTGAACGAAATAATGGA

Cucsa.097760-r ------------------------------------------------------------

Cla008358-r ------------------------------------------------------------

MELO3C002189-r ------------------------------------------------------------

CSPI01G07780-r ------------------------------------------------------------

CmaCh13G009390-r AGATAAAACTTCATCTAGACTAGGCAACCCTTTTCTTGTAGTTAAGTGACATTCGCTAGG

Cucsa.097760-r ------------------------------------------------------------

Cla008358-r ------------------------------------------------------------

MELO3C002189-r ------------------------------------------------------------

CSPI01G07780-r ------------------------------------------------------------

CmaCh13G009390-r ATATGATAGATTGGAAGTTTGTCAAGAAGGTATATGTCCCCATTCAATCCCACACGTGCC

Cucsa.097760-r ------------------------------------------------------------

Cla008358-r ------------------------------------------------------------

MELO3C002189-r ------------------------------------------------------------

CSPI01G07780-r ------------------------------------------------------------

CmaCh13G009390-r ATTTCAAAATTAAGAATTGAAATTGGCAAATGTGGAGATATGGTTTTTAGTCATTTACTT

Cucsa.097760-r ------------------------------------------------------------

Cla008358-r ------------------------------------------------------------

MELO3C002189-r ------------------------------------------------------------

CSPI01G07780-r ------------------------------------------------------------

CmaCh13G009390-r GAAAAATTAGTGGATGATGTATTAAGACAATAGCACGGAAAACATATATTCTAGTTTCAT

Cucsa.097760-r -------------------------------------------

Cla008358-r -------------------------------------------

MELO3C002189-r -------------------------------------------

CSPI01G07780-r -------------------------------------------

CmaCh13G009390-r ATATTGACTTGAGAATGCATCATTACTTTATGGAAAGAAAAAA

**Figure S2 Alignment of exons of *alcohol dehydrogenase-like 6* genes**

cucumber (Cucsa.097760-r, Chinese long 9930; CSPI01G07780-r, Wild cucumber PI 183967)), melon (MELO3C002189-r), water melon (Cla008358-r) and pumpkin (CmaCh13G009390-r)

**
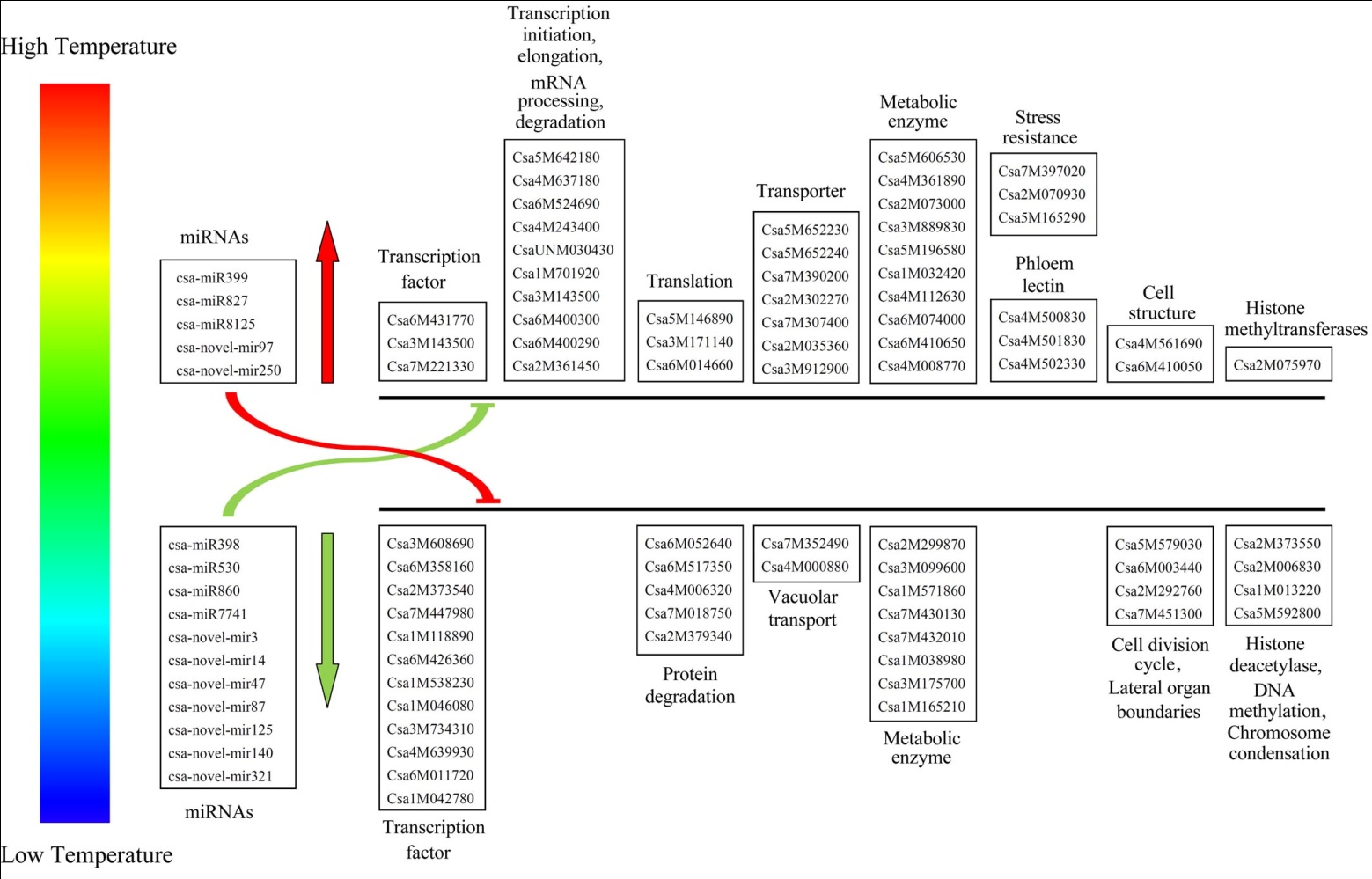
**

**Figure S3 Schematic diagram of the miRNA-target network modulating the plant adaptation to temperature changes**

based on functional annotations (computer predicted targets) of the temperature affected miRNAs
